# Roles of airway smooth muscle dysfunction in chronic obstructive pulmonary disease

**DOI:** 10.1186/s12967-018-1635-z

**Published:** 2018-09-26

**Authors:** Furong Yan, Hongzhi Gao, Hong Zhao, Madhav Bhatia, Yiming Zeng

**Affiliations:** 1 Center for Molecular Diagnosis and Therapy, Second Affiliated Hospital of Fujian Medical University, Quanzhou, Fujian China; 2 Department of Pathology and Biomedical Science, University of Otago, Christchurch, New Zealand; 3 Department of Pulmonary and Critical Care Medicine, Respiratory Medicine Center of Fujian Province, Second Affiliated Hospital of Fujian Medical University, Quanzhou, Fujian China

**Keywords:** COPD, ASM dysfunction, Proliferation, Phenotype shift

## Abstract

The airway smooth muscle (ASM) plays an indispensable role in airway structure and function. Dysfunction in ASM plays a central role in the pathogenesis of chronic obstructive pulmonary disease (COPD) and contributes to alterations of contractility, inflammatory response, immunoreaction, phenotype, quantity, and size of airways. ASM makes a key contribution in COPD by various mechanisms including altered contractility and relaxation induce by [Ca^2+^]_i_, cell proliferation and hypertrophy, production and modulation of extracellular cytokines, and release of pro-and-anti-inflammatory mediators. Multiple dysfunctions of ASM contribute to modulating airway responses to stimuli, remodeling, and fibrosis, as well as influence the compliance of lungs. The present review highlights regulatory roles of multiple factors in the development of ASM dysfunction in COPD, aims to understand the regulatory mechanism by which ASM dysfunctions are initiated, and explores the clinical significance of ASM on alterations of airway structure and function in COPD and development of novel therapeutic strategies for COPD.

## Background

**Fig. 1:**
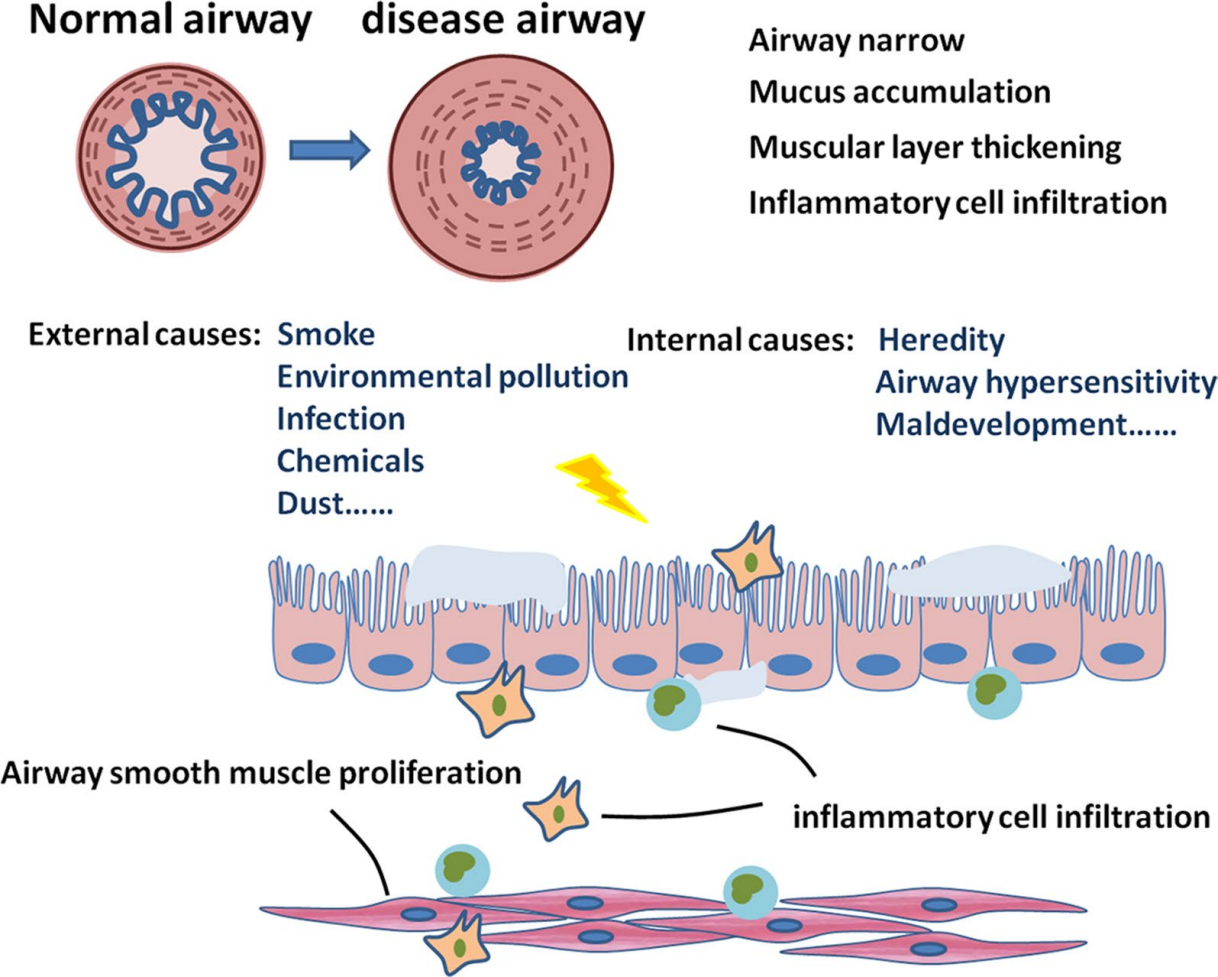
ASM dysfunction plays an important role in the pathogenesis of COPD. The pathogenesis of COPD has been divided into external and internal causes. Airway remodeling is a vital feature of COPD. Dysfunctions in ASM function are central to the pathogenesis of airway remodeling. Changes of ASM include alteration of contractility, inflammatory response, immunoreaction, phenotype, quantity and size

Chronic obstructive pulmonary disease (COPD) is a major cause of morbidity and mortality of patients with lung diseases, characterized by persistent airflow obstruction, with an enhanced inflammatory response in lungs and airways. COPD was the third leading cause of death in China with more than 0.9 million deaths in 2013 [1]. Overwhelming majority of patients had varying degrees of airway remodeling and narrowing. The number of airway smooth muscle (ASM) of COPD patients with the GOLD standard in grade 3 and 4 increased by nearly 50% [2], which was negatively correlated with lung function in COPD patients. A variety of cellular mediators and pathways contribute to the pathogenesis of COPD, evoking a large number of airway and lung dysfunctions [3]. Cigarette smoking is the major cause for COPD, while the pollution exposure such as biomass cooking, heating, and exhaust gas, are also very important factors [4]. In addition, environmental alterations, genetic abnormalities, abnormal lung development and accelerated aging also contribute to the development of COPD [3]. The limitation of airflow as the principal feature of COPD is progressive and not completely reversible [5] and is caused by airway remodeling, loss of small airways, and emphysema [6]. Of those, the airway remodeling and inflammation are considered as the major factors resulting in irreversible airflow limitation [2]. The airway remodeling in COPD includes mucosal hyperproduction, epithelial hyperplasia and metaplasia, increased basement membrane thickness, and connective tissue over-deposition, as well as increased mass of ASM (Fig. 1).

Smooth muscle cells are a crucial component of airway for the contractile function and contributions to the production of inflammatory factors, proteases and growth factors [7]. The altered contractile function and mass of ASM leads to airway inflammation, hyperresponsiveness, and remodeling [8], which are key defining features of COPD. Recent studies demonstrated that changes in other ASM functions like oxidant/antioxidant imbalance, inflammatory secretion and metabolic disorder also contribute significantly to COPD pathophysiology. For example, the nicotinamide adenine dinucleotide phosphate oxidase 4 (NOX4) expression was up-regulated in ASM during COPD, and strongly related with smoking [9]. ASM in COPD showed imbalance and accumulation in some glycolytic products like lactate, glutamine, fatty acids and amino acids [10]. Moreover, ASM also increases the release of inflammatory mediators to play an important role in many aspects of COPD pathogenesis. The present review aims to briefly overview how the abnormal contractility of ASM contributes to the airway remodeling in COPD, ASM-dominated airway inflammation occurs, ASM is involved in the development of local and systemic immune response to challenges, and the ASM mass and phenotype changes during COPD. Altered ASM plays an important role in the pathogenesis of COPD and contributes to the severity of the disease.

## ASM contractility

**Fig. 2:**
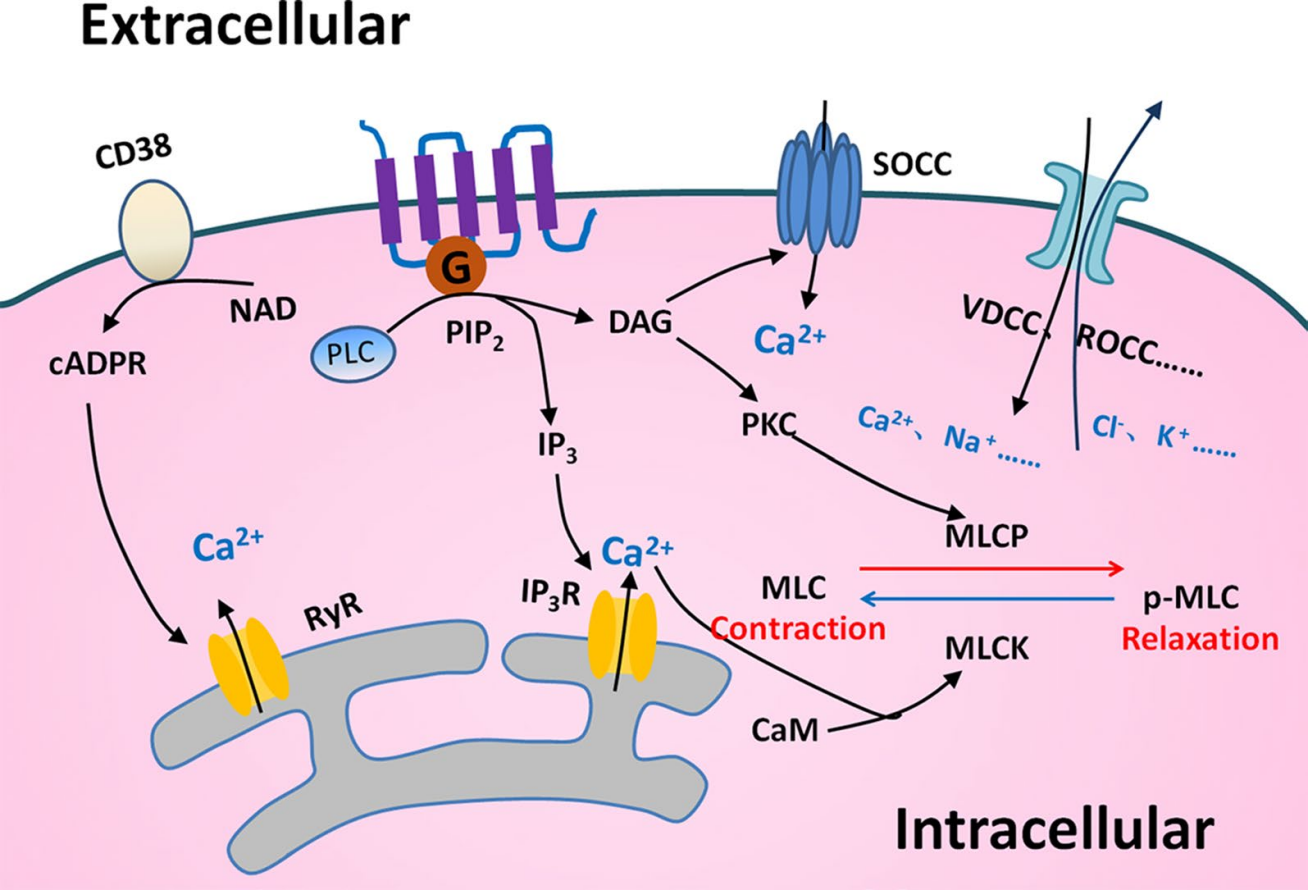
Mechanisms of contraction in ASM. Many regulatory mechanisms in ASM to control the contraction and relaxation are well recognized. The ectoenzyme CD38 could produce the second messenger cyclic ADP ribose (cADPR), which causes Ca^2+^ release through ryanodine receptor (RyR) channels from sarcoplasmic reticulum (SR). The G-protein coupled receptor (GPCR)-based pathway activates phospholipase C (PLC) and breaks up phosphatidyl diphosphate inositol 2 (PIP_2_) into inositol trisphosphate (IP_3_) and diacylglycerol (DAG). Intracellular Ca^2+^ binds to intracellular calmodulin (CAM) to alter the phosphorylation status of myosin light chain (MLC) and regulates ASM function. Depletion of Ca^2+^ in SR calcium stores induces Ca^2+^ influx through store-operated Ca^2+^ channel (SOCC). There are other mechanisms that take part in regulating the intracellular ion concentration of ASM and finally affect the contraction and relaxation of them

Airway smooth muscle plays a vital role in the regulation of bronchomotor tone and in the control of the airway caliber, by which COPD patients may have an exaggerated progression of ASM contractility. A variety of regulatory mechanisms are involved in the ASM contraction, leading to the occurrence of airway hyperresponsiveness. Key mechanisms include G-protein coupled receptor-based pathways [11] such as Gq and Gi-dependent signaling, nonselective cation channels especially transient receptor potential channels [12], and store-operated calcium channel [13] (Fig. 2). ASM maintains a balance between the airway hyper-reactivity and bronchodilation when it response to stimuli [14]. The abnormal regulation of contraction and relaxation in ASM leads to the development of disease. For example, the ASM contractile activity altered in lung fibrotic processes, leading to the abnormality of the mechanical properties of airway and contributing to the pathogenesis of COPD.

A number of factors are involved in the dysregulated mechanism of ASM tone in COPD, e.g. ion channels reside in ASM cell plasma membrane, including voltage-gated channels, receptor- and store-depended channels which are consisted of a variety of transient receptor potential (TRP) channels, stretch-activated channels, and Ca^2+^-dependent K^+^ channels [15]. Of these, TRPV4, the subtype of TRP channels plays an important role in the development of COPD-related airway activities. The bronchial epithelial cells serve as the primary receptor to be initially stimulated by external challenges and as the barrier to separate ASM from the air. When ASM was exposed to hypotonic airway space liquid and gas, the airway epithelial cell barrier dysfunction occurred in patients with COPD. The activation of TRPV4 in ASM can trigger the ASM contraction, damaged epithelial cells induce the loss of ASM constrictive capability for releasing NO, and the airway can be consistently contracted in COPD patients under hypotonic stimulation [16]. TRPA1, another subtype of TRP channels are a determinant of susceptibility in the development of COPD, mobilize Ca^2+^ influx in ASM upon activation, and regulate airway contraction via release of neuropeptides, e.g. calcitonin related polypeptide alpha and substance P [17]. G protein-coupled receptors especially Gq-coupled pathways which mainly affects the contractility of ASM could be used as a major drug targets for COPD through elevating Ca^2+^ levels by activating the phospholipase C-inositol 1,4,5-trisphosphate pathway in ASM. Conversely, Gs-coupled pathway is important for airway dilation, particularly through β_2_-adrenoceptor action enhancing cAMP [11, 18, 19]. Recent studies suggests that the interactions between taste 2 receptor member 1-bronchoconstrictor and G protein-coupled receptors may contribute to airway contractility and its agonist such as caffeine can influence the actin polymerization of ASM in COPD [20]. Local levels of thromboxane 2 are elevated in the airway of patients with COPD [21]. It indicates that endogenous bronchoconstrictors can be one of the major reasons responsible for the high intension of ASM and the airway hypercontraction in COPD through the coupling between the thromboxane-prostanoid receptor with Gq-coupled pathway and through the PLC/IP_3_/Ca^2+^ pathway.

## ASM-dominated inflammatory responses

The over-contraction of ASM induced through the activation of multi-kinases also plays a key role in the development of chronic inflammation and airway remodeling in patients with COPD [18]. When the airway is exposed to viruses, bacteria, or other pathogens, a number of airway and lung cells are provoked and over-reacted to resulting in the development of the airway inflammation in COPD. ASM cells are activated by inflammatory signals from airway epithelial cells, leukocytes, and others, and then induce the secondary inflammation by producing inflammatory mediators. A recently study has shown that ASM cells can act as producers of pro-and anti-inflammatory mediators (e.g. cytokines, chemokines, growth factors) to regulate the local immune environment and influence proliferation, migration, and apoptosis of other resident cells [22].

Airway smooth muscle can also act as a source of extracellular matrix proteins, leading to structural changes and remodeling of the airway and alveoli. ASM cells produce monocyte chemoattractant protein-1, C-X-C motif chemokine ligand 10, interleukin (IL)-6, IL-8, IL-1β and eotaxin [23–25], through the selective expression of most Toll-like receptors isoforms. In human ASM, the active or inactivated virus could cause the release of IL-5 and IL-1β from human ASM cells, probably through intercellular adhesion molecule-1, since such production is prevented by treatment with the antibody against intercellular adhesion molecule-1 [26]. IL-1β and tumor necrosis factor-alpha (TNFα) can modulate inflammatory responses during the exacerbations of COPD.

In addition, the abnormality of the mitochondrial function occurs within ASM cells from patients with COPD, evidenced by the excessive production of reactive oxygen species. The production of inflammatory responses and mitochondrial dysfunction are the part of cell self-defenses and the secondary inducer of tissue inflammation and injury to activate and drive the innate immune system in the pathogenesis of COPD [27]. β-catenin as a cellular homeostasis regulator can control the cell division and differentiation and take part in the inflammatory processes of chronic airway disease, primarily through the co-action with nuclear factor-κB in human ASM and with the nuclear cofactors CREB-binding protein (CBP) and its homologue p300 [28].

## ASM-associated immunoreactions

The tissue forming cells, especially of ASM, play an important role in the immune reaction during the development of COPD by recognizing environmental factors through immune globulin receptors or/and through non-immune systems [29]. ASM may amplify or dampen responses to pathogens by releasing mediators, interacting with recruiting immune cells, and increasing the responsiveness of ASM to stimuli. The immune-regulatory capacity of ASM also includes its response to cytokines such as IL-1β, TNF-α and IFN-γ. During such process, ASM can initiate the over-expression of cell adhesion and co-stimulatory molecules, which attract the recruitment of multiple immune cells into airways and modulate responses to irritants. ASM-produced cytokines can induce the hyperplasia and modulate immune cell function. ASM-origin TNF-α stimulates eosinophilia and neutrophilia in the airway, results in the maturation and differentiation of structural tissues, and leads to the over-expression of cell adhesion molecules and T cell activation [30]. On the other hand, external TNF-α can promote roles of ASM in the infiltration and adhesion of activated immune cells and interaction with and adhesion to inflammatory cells [31–33]. ASM can also communicate with airway epithelial cells which can be activated to produce the inflammatory mediators, resulting to the secondary systemic inflammation [34]. ASM can activate immune cells like T lymphocytes and dendritic cells (DCs) by producing thymic stromal lymphopoietin (TSLP), and promote the maturation of T cell phenotype shift [35, 36]. TSLP as a proinflammatory cytokines has been linked to chronic airway diseases. Recent studies have shown that ASM are capable in expressing TSLP in vitro and in vivo, and the enhanced production of it, in turn, creates an inflammatory microenvironment to activating local inflammatory response and aggravating airway remodeling, which is associated with COPD. TSLP-activated DCs in vitro play an important role in promoting the differentiation of Th17 cells with the central memory T cell phenotype [37]. They also induce a unique Th2 cell phenotype that may produces a large amounts of TNF, but little or no IL-10, which different with normal Th2 cells [38].

## Change of ASM remodeling

**Fig. 3:**
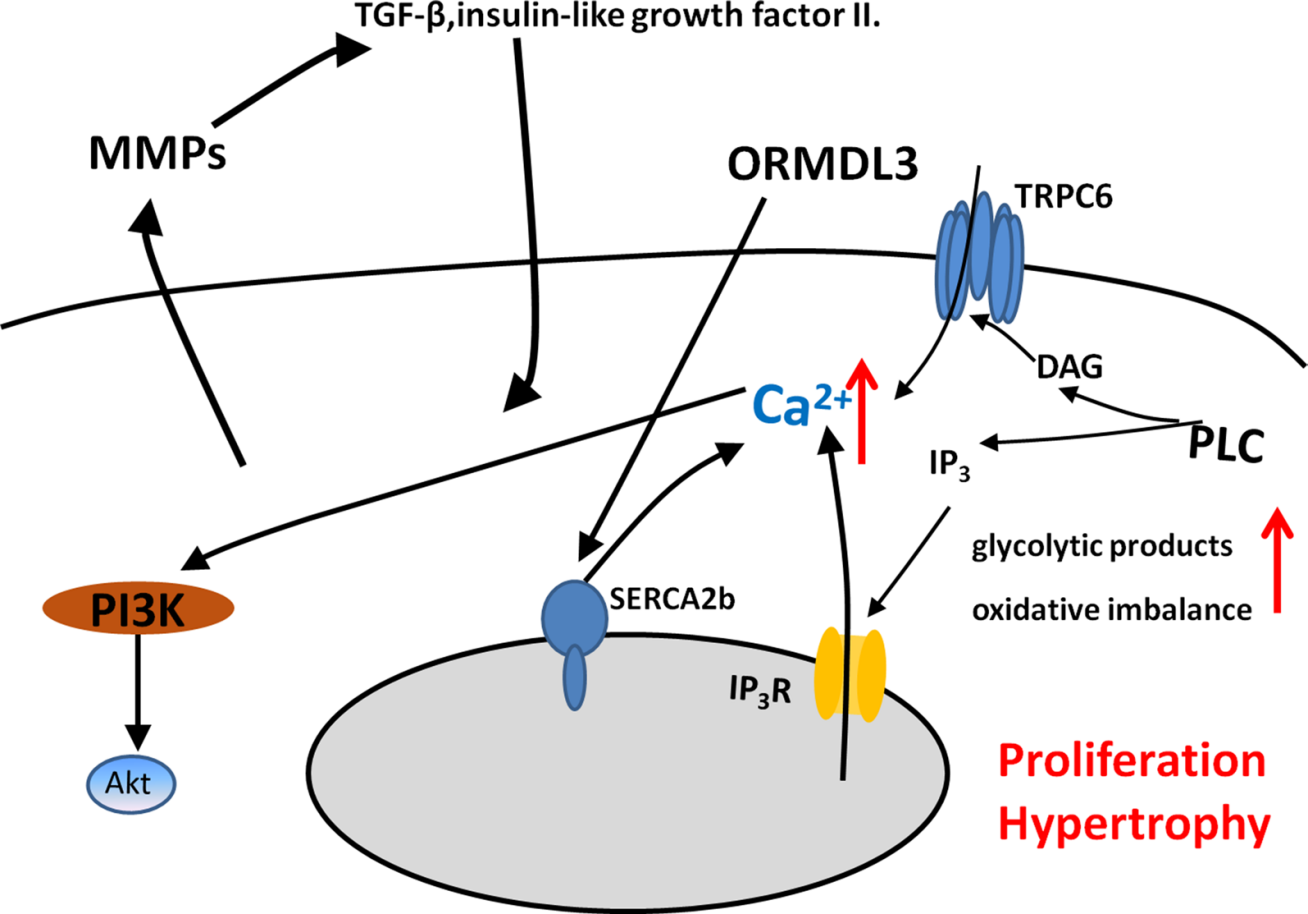
Molecular mechanisms of airway smooth muscle proliferation and hypertrophy in COPD. The proliferation and hypertrophy of ASM is mainly caused by the stimulation of inflammatory factors, the increase of intracellular calcium concentration, the antioxidant imbalance, and the accumulation of metabolic products

The hypertrophy and hyperplasia of ASM can be negatively correlated with lung function and positively with the severity of airway thickening [2] (Fig. 3). Airrway remodeling is usually accompanied with ASM proliferation. ASM is an important source of matrix metalloproteinases (MMPs). Zinc-dependent proteolytic enzymes produced by ASM play a major regulatory role in matrix turnover, remodeling, and angiogenesis of airways in COPD [6]. Of these, MMPs induce smooth muscle proliferation by release of immobilized growth factors, such as TGF-β, and insulin-like growth factor II. Altered ASM are considered as a distinct metabolic phenotype of COPD. The damaged energy balance and accumulation of the glycolytic products, e.g. lactate, glutamine, fatty acids, and amino acids, increased biosynthesis and redox imbalance in ASM of patients with COPD, and supported ASM proliferation and survival [10]. The cigarette smoke extract (CSE) is the most dangerous environmental factor that promotes the proliferation of ASM in COPD, and it happens in connection with up-regulated expression of calreticulin and down-regulated expression of C/EBPα [39]. Moreover, CSE enhances proliferation of ASM by up-regulation of PCNA and Cyclin E [40]. Calcium influx in ASM by α7 nAChR-PI3K/Akt-TRPC6 pathway contributes to the proliferation process of ASM primarily associated with cigarette smoke [41]. Hypertrophy of ASM is also an important mechanism of airway remodeling. The elevated expression of NOX4 in ASM of small airway may increased the volume of ASM [42]. Up-regulation in orosomucoid-like 3 (ORMDL3) expression can stimulate ASM proliferation, hypertrophy and contractility through enhancing Ca^2+^ flux induced by increased sarcoplasmic reticulum Ca^2+^ ATPase 2b (SERCA2b) expression [43]. The ASM remodeling has become an intractable problem in COPD, because of its irreversible changes and multiple mechanism regulation.

## Phenotype shift

**Fig. 4:**
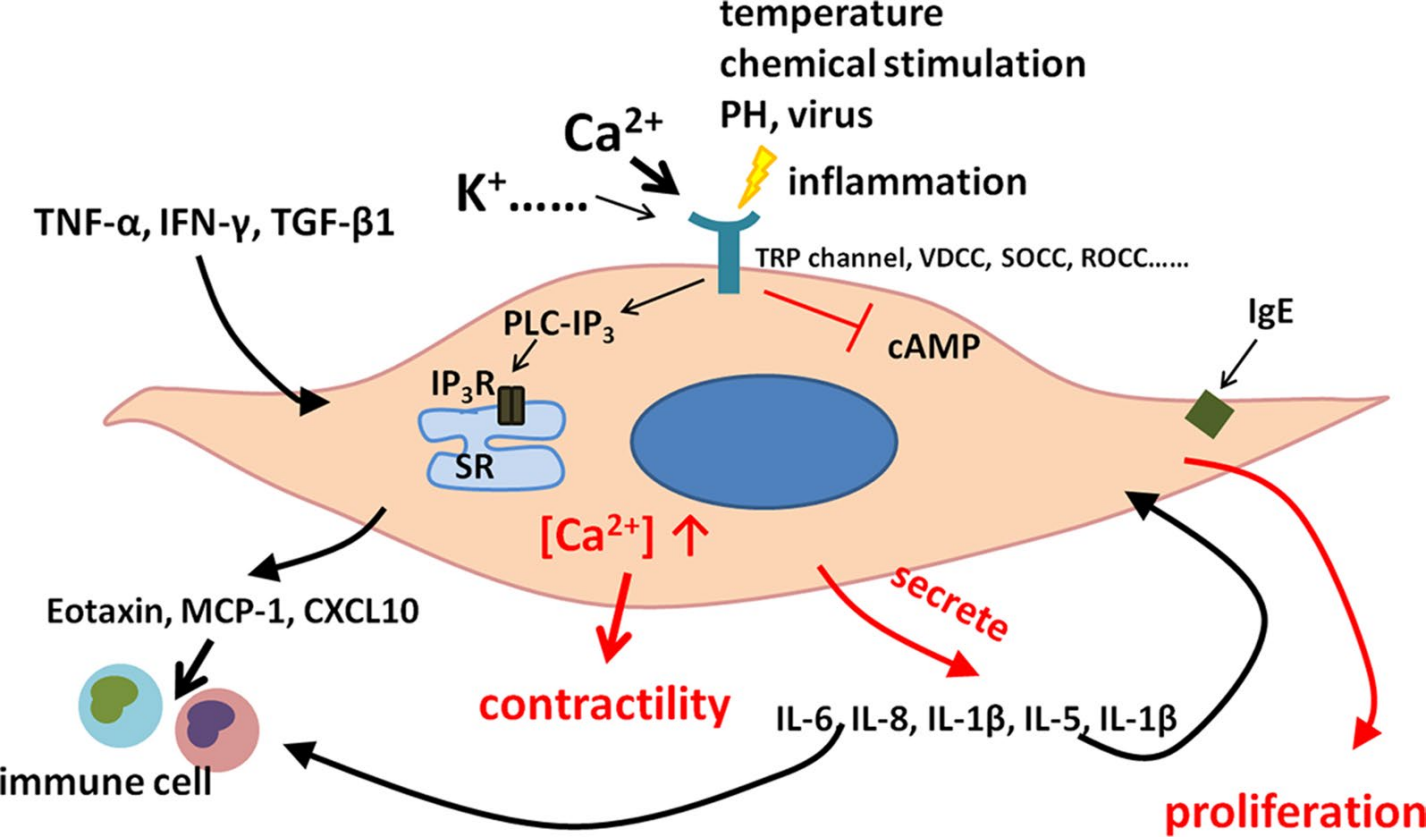
Molecular mechanisms of airway smooth muscle function changes in COPD. The ASM in patients with COPD can act as a producer of pro-and anti-inflammatory mediators (e.g. cytokines, chemokines, growth factors), to regulate the local immune environment and influence proliferation, migration, and apoptosis of other resident cells

The ASM phenotype shift is characterized by reversible switching between contractile and proliferative phenotype, of which such shift altered obviously in COPD. It has been demonstrated that ASM cells switch from a contractile to a proliferative phenotype are usually accompanied with reduced K_Ca_1.1 channels and enhanced K_Ca_3.1 channels. Up-regulated K_Ca_3.1 channels caused the increased expression of contractile phenotypic marker proteins and induced ASM migration and proliferation. Blockade of K_Ca_3.1 channels is considered as a therapeutic target in COPD [44]. Long non-coding RNAs (lncRNAs) are associated with ASM phenotype in COPD. The expression of some lncRNAs in healthy ASM cells was increased after stimulation with proliferation inducer [45]. Muscarinic receptors are activated to enhance functional effects of TGF-β1 in ASM, underpin ASM remodeling in COPD [46]. Smoking and local inflammation could directly lead to ASM proliferation through transformation from contractile ASM phenotype into proliferative phenotype in airway remodeling in COPD, which depends on phosphorylation of ERK 1/2 and p38 MAP kinase and downstream mitogenic signaling [47] (Fig. 4).

## Regulation of TRP channels in the function of ASM

Adjustment in intracellular calcium concentration ([Ca^2+^]_i_) homeostasis directly affects ASM multiple functions. Several members of TRP channels is calcium permeable cation channels and are important for calcium signal transduction in many cell types of respiratory pathophysiology. The TRP channels superfamily can be divided into seven subfamilies based on sequence homology: TRPA (ANKTM1), TRPC (canonical), TRPM (melastatin), TRPML (mucolipidins), TRPN (NompC), TRPV (vanil) and TRPP (polycystin) [48]. Transient receptor potential canonical (TRPC) channels belong to the TRP superfamily and most of them expressed in ASM [49, 50]. They may mediated changes in [Ca^2+^]_i_ and induced ASM abnormal contraction, proliferation, hypertrophy, as well as secretion of inflammatory mediators [41, 51]. Calcium influx in ASM stimulating by cigarette smoke activates α7 nAChR-PI3K/Akt-TRPC6 pathway and contributes to the proliferation process of ASM [41]. Deletion of TRPC6 has both elevated in airway resistance and arterial pressure. TRPC3 involved in both TNF-α and acetylcholine induced Ca^2+^ influx in ASM [51] and overexpression of it may contributes to hyperresponsiveness and remodeling in ASM [52]. TRPV4 as an osmolarity sensor is expressed in ASM and may induced Ca^2+^ influx when stimulated by hypotonic stimulation [53]. In recent years, TRPA1 has also been found to be highly expressed on the membrane of ASM and can be activated by temperature and exogenous irritants, leading to airway inflammation and high reactivity [54]. TRP channels were widely involved in the physiological and pathological changes of various respiratory systems, and have become a new target for the treatment of COPD.

## ASM in the development of novel therapeutic approaches for COPD

Airway smooth muscle is the important part in the treatment of airways disorders, for which the targeted therapies are the mainstay in current management of COPD, although controversies are full in the use of those drugs. Potential new targets related to ASM are emerging, despite a long time needed to discover novel classes of COPD drugs. Pathophysiological mechanisms of ASM significantly contribute to the occurrence and progression of COPD. In order to develop therapeutic strategies, key pathological mechanisms and special clinical targets need to be furthermore identified. Current therapies for ASM to treat COPD primarily is bronchodilators through preventing or reversing abnormal shrinkage enhancement of ASM. Most bronchodilators targets in GPCR ligands and work by either promoting relaxant signaling or inhibiting contractile signaling. Airway changes in COPD are also affected by structural modification, which induced by phenotypic shift and increased secretion of cytokines, but currently available therapies for COPD rarely targeted on it. The use of inhibitors of K_Ca_1.1 channels is used to treat COPD by suppressing ASM phenotype shift to proliferative type [45]. Oxidative stress is crucial in the pathogenesis of COPD, but current treatment do not specifically target oxidative stress. Imbalance of oxidative stress responses in ASM have been shown in patients with COPD and contributed to airway inflammatory reaction and influenced ASM functions, probably through mitochondrial dysfunction. Targeting treatment with the mitochondria-targeted antioxidant leads to a reduction of the increased the secretion functions and reduce proliferation of ASM from patients with COPD [27]. Inhibition of NOX4 expression was markedly slowing COPD progress by modulating ROS production in ASM [9]. Sul-121 is a novel compound with anti-oxidative capacity and can effectively inhibit airway inflammation and airway hyperresponsiveness in COPD models by reducing intracellular reactive oxygen species production in ASM [55]. Leptin receptor increased expressed on ASM of COPD and has be found inhibited ASM proliferation, migration and eotaxin production through stimulating ASM to secrete prostaglandin E2 [56]. Retinoic acid can inhibit ASM migration by blocking the PI3K/Akt pathway [57]. There are huge research prospects on the treatment of COPD. The main therapies for ASM to treat COPD have been summarised in Table 1.

**Table 1:** Therapeutic approaches targeted on ASM

ASM function changes	Type	Action mechanism	References
Contraction and relaxation	Anticholinergics	Inhibit the M2 and M3 subtypes of muscarinic acetylcholine receptors	[59]
	β_2_-adrenoceptor agonists	Stimulation of adenylyl cyclase and subsequent activation of the cAMP-dependent signaling	[60]
	Glucocorticoids	Reduce ASM contraction induced by histamine, bradykinin and acetylcholine	[61]
Proliferation and migration	K_Ca_1.1 channels inhibitor	Suppressed phenotype shift to proliferation	[44]
	β_2_-adrenoceptor agonists	Blocked chemoattractants induced ASM migration	[62]
	Leukotriene receptor antagonists	Leukotriene B4 markedly induced proliferation of human ASM cells	[63]
	Antioxidant, glucocorticoids, ASK1 inhibitor	Inhibited TGF-β-induced ASM cell proliferation	[27, 64, 65]
	Leptin	Stimulating ASM to secrete prostaglandin E2	[56]
Secretion	Antioxidant	Inhibited CXCL8 release from ASM	[27]
		Reduced intracellular reactive oxygen production in ASM	[55]
	Vitamin D	Inhibits secretion of RANTES	[66]

## Conclusion

Alterations of ASM morphology and function are considered as the determinant of the airway function and contribute to the severity of COPD. Different factors produced by ASM can induce inflammation, proliferation, apoptosis, and differentiation of ASM per se and also epithelial cells and immune cells. ASM can serve as a receptor cell to be stimulated and activated by systemic inflammation to develop hypertrophy and hyperplasia in COPD. More importantly, ASM can act as an initiator of secondary inflammation to activate other cells. Thus, ASM plays critical and irreplaceable roles in the development of airway inflammation and remodeling during COPD. Current researches on COPD often using stimulant likes cigarette, lipopolysaccharide and protease either individually or jointly to construct models. A low dose of rhinovirus infection in patient with COPD would reproduce the features of COPD exacerbations and be know as a human model of COPD exacerbations [58]. Although these models have provided great help for clinical and scientific research on COPD, there are still some weakness on them for ASM dysfunction researches in COPD because of the complex mechanism. Due to limited understanding of mechanism and lack of appropriate models, the importance of ASM in COPD has not been given sufficient attention. To elucidate the etiology and improve treatment of COPD, further research is needed. As we find out more about the ASM dysfunction in COPD, we will find more options for specific therapeutic method to improve the clinical symptoms and the quality of life in patients with COPD.

## Abbreviations

ASM: airway smooth muscle
COPD: chronic obstructive pulmonary disease
[Ca^2+^]_i_: intracellular free calcium concentration
CSE: cigarette smoke extract
EBP-α: enhancer-binding protein-α
IFN-γ: interferon-γ
IL: interleukin
IP_3_: inositol tris-phosphate
MMPs: matrix metalloproteinases
NOX4: nicotinamide adenine dinucleotide phosphate oxidase 4
PLC: phospholipase C
TGF-β: transforming growth factor-β
TNF-α: tumor necrosis factor-α
TRP: transient receptor potential
